# Transcriptome Sequencing Analyses between the Cytoplasmic Male Sterile Line and Its Maintainer Line in Welsh Onion (*Allium fistulosum* L.)

**DOI:** 10.3390/ijms17071058

**Published:** 2016-07-01

**Authors:** Qianchun Liu, Yanping Lan, Changlong Wen, Hong Zhao, Jian Wang, Yongqin Wang

**Affiliations:** 1 Beijing Vegetable Research Center, Beijing Academy of Agriculture and Forestry Sciences/Key Laboratory of Biology and Genetic Improvement of Horticultural Crops (North China), Ministry of Agriculture, Beijing 100097, China; lqc072150@126.com (Q.L.); wenchanglong@nercv.org (C.W.); zhaohong@nercv.org (H.Z.); wyqty@sohu.com (J.W.); 2 Institute of Agricultural Integrated Development, Beijing Academy of Agriculture and Forestry Sciences, Beijing 100097, China; lanyanping2000@126.com

**Keywords:** Welsh onion, transcriptome sequencing, cytoplasmic male sterility (CMS)

## Abstract

Cytoplasmic male sterility (CMS) is important for exploiting heterosis in crop plants and also serves as a model for investigating nuclear–cytoplasmic interaction. The molecular mechanism of cytoplasmic male sterility and fertility restoration was investigated in several important economic crops but remains poorly understood in the Welsh onion. Therefore, we compared the differences between the CMS line 64-2 and its maintainer line 64-1 using transcriptome sequencing with the aim of determining critical genes and pathways associated with male sterility. This study combined two years of RNA-seq data; there were 1504 unigenes (in May 2013) and 2928 unigenes (in May 2014) that were differentially expressed between the CMS and cytoplasmic male maintainer Welsh onion varieties. Known CMS-related genes were found in the set of differentially expressed genes and checked by qPCR. These genes included F-type ATPase, NADH dehydrogenase, cytochrome c oxidase, etc. Overall, this study demonstrated that the CMS regulatory genes and pathways may be associated with the mitochondria and nucleus in the Welsh onion. We believe that this transcriptome dataset will accelerate the research on CMS gene clones and other functional genomics research on *A. fistulosum* L.

## 1. Introduction

As an important vegetable crop, The Welsh onion (*Allium fistulosum* L.) is cultivated worldwide from tropical Asia to Northeast Asia. Because it is rich in propylene sulfide having bactericidal and anti-inflammatory effects, the Welsh onion has also been used as an herbal medicine for diverse diseases, such as febrile disease, headache, diarrhea, abdominal pain, eye-related disorders, and habitual abortion [1]. The Welsh onion is a cross-pollinated crop, and heterosis has been extensively applied in breeding [2]. Artificial emasculation is difficult in this species because of its small flower. Therefore, cytoplasmic male sterility (CMS) plays a crucial role in heterosis breeding.

CMS is a phenotypic trait that is widespread among plants and results in the inability of a plant to produce viable pollen [3], using CMS lines to obtain F_1_ hybrids, which is the preferred method in heterosis breeding. The CMS plants are usually obtained by natural populations [4,5], crossed by different genera and species plants [6,7], physical and chemical mutagenesis [8], and plant protoplast fusion [9,10]. As early as 1972, Nishimura and Shibano discovered spontaneous CMS of onion in Japan [11]. Thereafter, Chinese researchers discovered the CMS line in the Welsh onion (*Allium fistulosum* L.) [12]. It has been proven to be an efficient way to obtain a heterosis hybrid, harboring important commercial benefit in the Welsh onion seed market [13,14,15].

Recently, the chromosomal location of the pollen fertility-restoring gene in *A. fistulosum* has been reported [15]. Furthermore, the CMS is closely linked to molecular markers. Restriction fragment length polymorphism (RFLP), rapid amplification of cDNA ends (RACE) and PCR-markers were used to identify cytoplasm types in the onion [16,17,18]. Subsequently, isozymes, random amplified polymorphic DNA (RAPD) and sequence characterized amplified region (SCAR) markers linked to the fertility restoring gene (*Rf*) locus were successfully developed [13]. However, the genetic gene or linked molecular marker of CMS in the Welsh onion has not been performed.

Some studies have shown that male-sterility-inducing factors are present in mitochondrial genomes [19], while others have reported that nuclear–mitochondrial interaction results in CMS [20,21]. This study, focusing on transcriptome sequencing comparative analysis of the CMS line and its maintainer line, may aid in screening the CMS-related genes and pathways in the Welsh onion and contribute to a better understanding of the CMS interaction of the nuclear and mitochondrial genomes in *A. fistulosum*.

## 2. Results

### 2.1. Sequencing and Transcriptome Assembly

To maximize the range of transcript diversity, a mixed RNA sample from three inflorescences in the flowering stage of the Welsh onion was prepared for RNA-seq using the Illumina HiSeq^TM^ 2000. After stringent quality assessment and data filtering, we generated 57.19 million read pairs that corresponded to 11.55 Gb of sequence data in May 2013, and 56.82 million read pairs that corresponded to 11.48 Gb of sequence data in May 2014 (Table 1). The Q30 percentages exceeded 85% in both years. In May 2013, using the Trinity de novo assembly program, the next-generation short-read sequences were assembled into 97,230 transcripts which had a mean length of 917.56 bp. A total of 52,126 unigenes with an average length of 755.69 bp were obtained. The N50 values of the transcripts and unigenes were 1419 and 1247 bp, respectively. In May 2014, there were 308,353 transcripts with a mean length of 1122.65 bp, and there were 70,360 unigenes with an average length of 879.72 bp. The N50 values of the transcripts and unigenes were 1813 and 1267 bp, respectively (Table S1). The longest transcript was taken as the sample unigene for data from each year.

The distribution curve for each sample was relatively flat in the randomly fragmented transcriptome. The number of sample sequences reached saturation under current conditions (Figure S1). The length distributions of the ORFs are shown in Figure S2. These results indicate that the sequencing quality and throughput were sufficient for the subsequent analyses.

### 2.2. Functional Annotation and Classification

**Figure 1:**
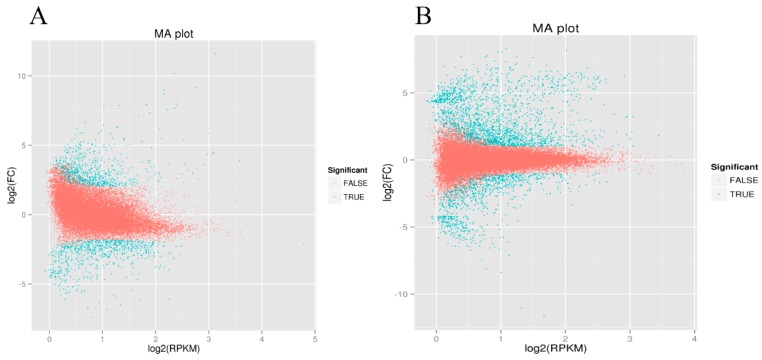
Differentially expressed genes in CMS vs. cytoplasmic male maintainer: (**A**) Whole-study overview of log-fold changes in gene expression in CMS vs. cytoplasmic male maintainer in May 2013; (**B**) Whole-study overview of log-fold changes in gene expression in CMS vs. cytoplasmic male maintainer in May 2014. The *x*-axis indicates the absolute expression levels (log_2_ RPKM (Reads Per Kilobase per Million mapped reads)). The *y*-axis indicates the log_2_-FC (fold changes) between the two samples. Genes for which differential expression is significant are shown as blue dots (Log_2_FC > 1 or < −1; FDR (False Discovery Rate) < 0.01).

Unigenes that had at least one significant match to an existing gene model in BLAST were determined using searches against the NCBI NR, Swiss-Prot, KEGG, GO, and COG. After screening all differentially expressed genes, we extracted identified information from the unigenes (Figure 1), and presented the overview of the results in Table S2.

**Figure 2:**
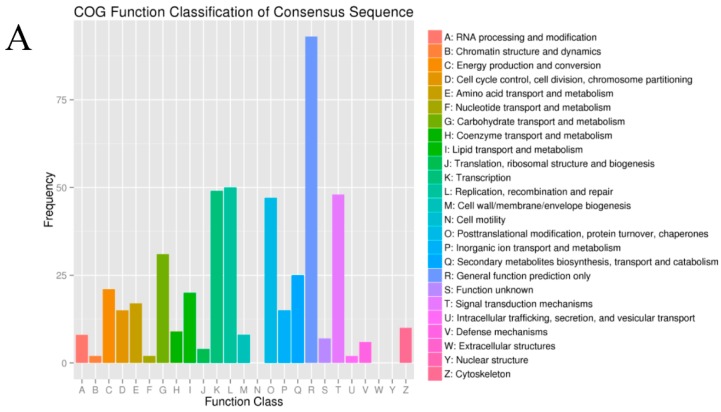

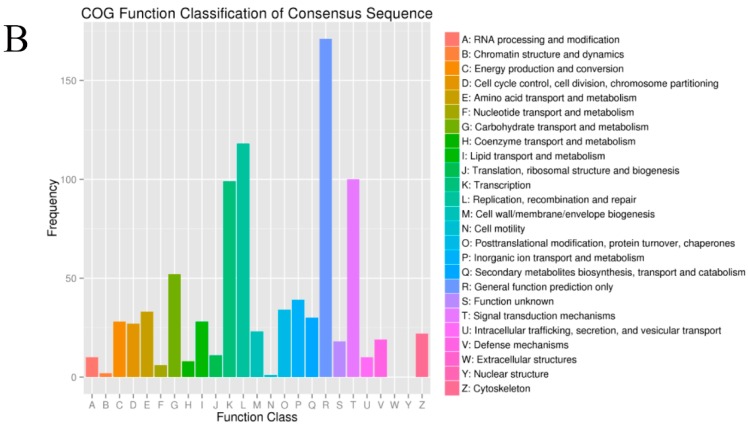
Cluster of Orthologous Groups (COG) categories of the unigenes: (**A**) sequencing data from May 2013; and (**B**) sequencing data from May 2014.

Using the COG functional classification, only 8326 of 52,126 (15.97%, May 2013) and 8201 of 70,360 (11.66%, May 2014) unigenes were annotated with COG in each assembly (Table S2). The unigenes were annotated in 22 (May 2013) and 23 (May 2014) COG categories (Figure 2). Among these, “the general function prediction” was the largest group, followed by “the replication, recombination and repair”, “transcription”, and “signal transduction mechanisms”, whereas the “Nuclear structure”, “Extracellular structures”, and “Cell motility” groups were the smallest.

### 2.3. Gene Ontology (GO) Classification

**Figure 3:**
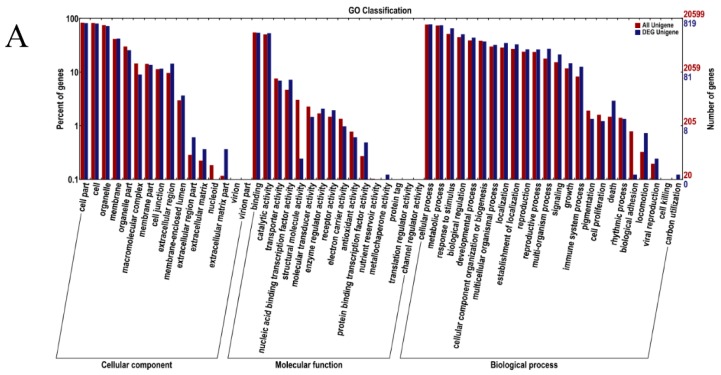

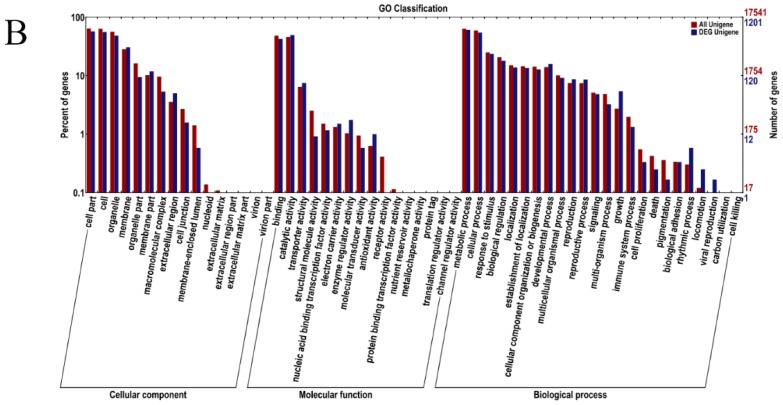
Gene Ontology Classification: (**A**) sequencing data from May 2013; and (**B**) sequencing data from May 2014. The unigenes are summarized into three main categories: cellular component, molecular function and biological process. The right side of the *y*-axis is the number of the genes; the left side of the *y*-axis is the percent of genes.

We applied GO terms to classify the functions of the assembled transcripts and describe gene products. A total of 20,599 unigenes (819 different unigenes in May 2013) and 17,541 unigenes (1201 different unigenes in May 2014) were assigned to 56 functional groups using GO assignment. The major different sub-categories within each of the three main categories (i.e., cellular component, molecular function and biological progress) of the GO classification are shown in Figure 3: there were five sub-categories in the cellular component cluster, i.e., “nucleus” (GO:0005634), “plasma membrane” (GO:0005886), “mitochondrion” (GO:0005739), “cytoplasmic membrane-bounded vesicle” (GO:0016023), and “membrane” (GO:0016020); there were four sub-categories in the molecular function cluster, i.e., “ATP binding” (GO:0005524), “binding” (GO:0005488), “metal ion binding” (GO:0046872) and “mitochondrial respiratory chain” (GO:0005746); and there were four sub-categories in the biological process cluster, i.e., “pollen tube growth” (GO:0009860), “phosphorylation” (GO:0016310), “oxidation-reduction process” (GO:0055114), and “response to abscisic acid” (GO:0009737).

### 2.4. Pathway Enrichment Analysis of DEGs (Differentially Expressed Unigenes)

**Figure 4:**
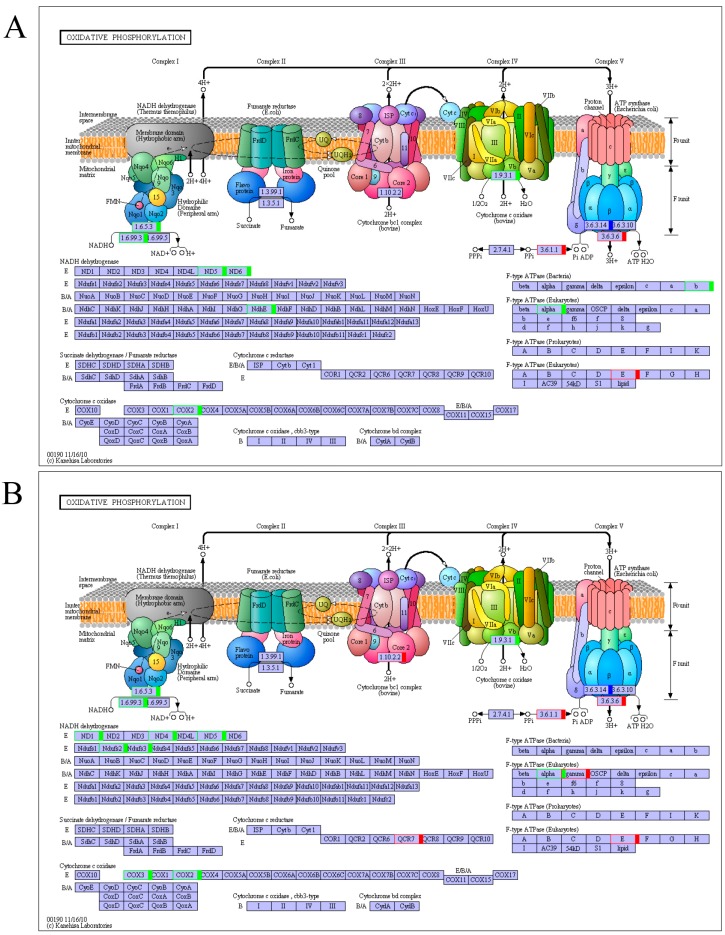
Welsh onion unigenes involved in oxidative phosphorylation pathways: (**A**) sequencing data from May 2013; and (**B**) sequencing data from May 2014. Blue boxes: enzymes specific to this species; Green boxes: down-regulated genes; Red boxes: up-regulated genes.

To categorize the biological functions of the DEGs, we performed a KEGG pathway enrichment analysis. The DEGs that significantly enriched pathways in May 2013 and May 2014 were the “plant-pathogen interaction” pathway, followed by the “oxidative phosphorylation” and “plant hormone signal transduction” pathways. In addition, pathways such as “starch and sucrose metabolism”, “glycerophospholipid metabolism”, and “protein processing in endoplasmic reticulum” composed a large component of the DEGs with pathway annotation (Table 2). In the above-mentioned DEGs with the best-represented KEGG pathways, there was consistency in enzyme regulation in the “oxidative phosphorylation” pathway between May 2013 and May 2014 (Figure 4). The enzymes “NADH: ubiquinone reductase (H^+^-translocating)”, “ND5” (NADH dehydrogenase 5), “COX2” (cytochrome-*c* oxidase 2), and “F-type ATPase α” (Eukaryotes) were related to down-regulated genes, whereas “inorganic diphosphatase”, “ATP phosphohydrolase” (H^+^-exporting), and “F-type ATPase E” (Eukaryotes) were related to up-regulated genes. “ATP phosphohydrolase” (H^+^-transporting) was related to both up- and down-regulated genes.

### 2.5. Analysis of CMS Related Genes in the Welsh Onion

**Figure 5:**
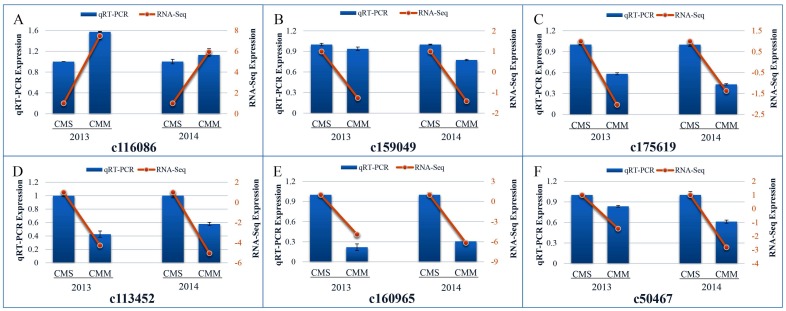
RT-qPCR and RNA-seq verification of differentially expressed candidate unigenes. CMS: Cytoplasmic male sterility; CMM: Cytoplasmic male maintainer. Data represent the average of three independent experiments. Error bars indicate the standard deviation (STDEV) for three biological replicates and three technical replicates in each experiment. (**A**–**F**) Different unigene IDs: (**A**) c116086; (**B**) c159049; (**C**) c175619; (**D**) c113452; (**E**) c160965; and (**F**) c50467.

This study utilized RNA-seq technology to explore CMS related genes between a CMS line and its maintainer line in the Welsh onion. All of the DEGs obtained from two years of RNA-seq data were investigated by BLAST in the NCBI database, and 559 unigenes were predicted to associate with CMS. Due to the DEGs that significantly enriched oxidative phosphorylation pathways in May 2013 and May 2014, the mitochondrial respiratory chain enzymes and enzyme complexes in oxidative phosphorylation pathways were important to CMS lines in other plants [22,23,24]. This study obtained six unigenes (IDs: c116086, c175619, c159049, c160965, c113452 and c50467) related to mitochondrial respiratory chain enzymes and enzyme complexes (Table 3), and they were significantly differentially expressed between the CMS line and the CMM line (Figure 5). These six unigenes were validated using RT-qPCR; the transcription expression of five unigenes was down-regulated in the cytoplasmic male maintainer line. Although most RT-qPCR results indicated smaller differences compared with the RNA-seq analysis, there was consistent expression. The unigenes functioned in “Energy production and conversion” (c175619, c159049) and “Posttranslational modification, protein turnover, and chaperones” (c173435) in the COG class annotation; they annotated to “Plasma membrane ATPase” (c116086), “Cytochrome c oxidase subunit 2” (c175619), “Cytochrome c oxidase subunit 3” (c159049), “Probable F-box protein” (c113452) and “Polygalacturonase inhibitor 1” (c50467) in the Swiss-Prot annotation; and, for the nt annotation, they were involved in the “*Cucumissativus* plasma membrane ATPase 4-like” (LOC101221564) (c116086), “*Hibiscus cannabinus* cultivar P3B cytochrome c oxidase subunit II (*cox2*) gene” (c175619), “*Allium cepa* cultivar saski cytochrome oxidase subunit 3 (*cox3*) gene, complete cds; mitochondrial” (c159049), and “*Allium cepa* cultivar saski NADH dehydrogenase subunit 1 (*nad1*) gene, partial cds; and ATPase α subunit (*atpA*) gene, complete cds; mitochondrial” (c160965).

## 3. Discussion

### 3.1. The Welsh Onion CMS Related Genes Were Enriched in the Oxidative Phosphorylation Pathway of the Mitochondrial Respiratory Chain

In this study, we profiled the Welsh onion transcriptome using the Illumina HiSeq 2000 platform, and classified the functions of the unigenes using the COG and GO classification and pathway enrichment analysis. A total of 1504 unigenes (in May 2013) and 2928 unigenes (in May 2014) were differentially expressed between the CMS line and its maintainer line in the Welsh onion.

In this study, we found many DEGs enriched in oxidative phosphorylation of the mitochondrial respiratory chain. The GO terms that identified the DEGs were related to CMS, i.e., “mitochondrion”, “mitochondrial respiratory chain”, “phosphorylation”, and “oxidation-reduction process”, and the cluster frequency of these DEGs was >5% (cluster frequency is the ratio of the number of DEGs that are annotated to this GO term to the number of DEGs that are annotated to all GO terms). The DEGs from the pathway analysis also showed many enzymes associated with the respiratory chain, and the regulation of numerous enzymes enriched in the “Oxidative phosphorylation” pathway, which is consistent in the RNA-seq data from both years.

In electron transfer system of mitochondrial respiratory chain, the respiratory chain enzymes and enzyme complexes (NADH dehydrogenase, cytochrome reductase, and cytochrome oxidase) play important roles in plants, their major function is to link electron transfer with proton translocation out of the mitochondrion, generating a transmembrane proton motive force that subsequently drives ATP synthesis by H^+^-ATPase [25]. In addition, cytochrome oxidase is the marker enzyme of the mitochondrial inner membrane with strong activity. Numerous studies have shown that cytochrome oxidase is relevant to CMS in plants [22,23]. In the pepper CMS line, a novel chimeric gene *orf 456*, was found to be inserted into the 3′-end of the *coxII* gene, and it may change the sterile characterization in the CMS line [22]. Luo et al. demonstrated that a new mitochondrial gene *WA352* encodes a protein that interacts with the nuclear-encoded mitochondrial protein COXII, thus causing premature tapetal programmed cell death and consequent male sterility [23]. In some studies, the CMS related candidate genes had close relationships with ATPase genes [2,11]. The F-type ATPase was proved to be embedded in the inner membranes of mitochondria, and this enzyme is a coupling factor in oxidative phosphorylation or photophosphorylation and plays an important role in energy conversion [24]. In this study, several key genes in the oxidative phosphorylation pathway were observed to be down-regulated by the RNA-seq approach; these gene-related enzymes were “NADH: ubiquinone reductase (H^+^-translocating)”, “ND5” (NADH dehydrogenase 5), “COXII” (cytochrome-c oxidase II), and “F-type ATPase α” (Eukaryotes). Two unigenes, c175619 and c159049, were annotated in “Energy production and conversion (COG)”; the unigene c116086 was identified as a plasma membrane ATPase (Swiss-Prot annotation), and the unigene c160965 was an ATPase α subunit (*atpA*) gene (nr annotation). In our previous study, the analysis of Two-Dimensional Electrophoresis results showed the ATPase α subunit was specifically expressed in the CMS line compared with the cytoplasmic male maintainer line (unpublished data). Based on the above research, we conjectured that CMS was closely related to the respiratory chain enzymes and enzyme complexes.

### 3.2. The Nuclear Cellular Component Function Genes Are Important to CMS in the Welsh Onion

In addition, more DEGs genes were obtained in the nucleus than in the mitochondrion: their cellular component functions (GO) were clustered in “nucleus” (Cluster frequency 25.42% in May 2013 and 13.43% in May 2014), “cytoplasmic membrane-bounded vesicle” (Cluster frequency 13.84% in May 2013 and 16.24% in May 2014), and “membrane” (Cluster frequency 15.68% in May 2013 and 12.70% in May 2014). In the COG functional classification, the “replication, recombination and repair” (Cluster frequency 10.22% in May 2013 and 13.27% in May 2014) and “transcription” (Cluster frequency 10.02% in May 2013 and 11.14% in May 2014) were the largest groups, and the processes of gene transcription mainly occur in the nucleus. Some research has reported that the sterility is caused by chimeric mitochondrial genes regulated by nuclear genes [26,27,28,29]. Thus, the nucleus may play an important role in the mechanisms of cytoplasmic male sterility. The results from Liu et al. suggest the CMS is the result of the nuclear–mitochondrial interaction [30]. In addition, Hong et al. suggested that nuclear–mitochondrial interaction may regulate pollen development and that the failure of sporogenous cell differentiation leads to sterility [31]. Therefore, we hypothesized that the Welsh onion male sterility regulatory genes and pathways are not only implicated in the mitochondria, but are also implicated in nuclear–mitochondria interaction. In addition, the identification of genes associated with male sterility in the nucleus remains to be further validated.

## 4. Methods and Methods

### 4.1. Sample Preparation

**Figure 6:**
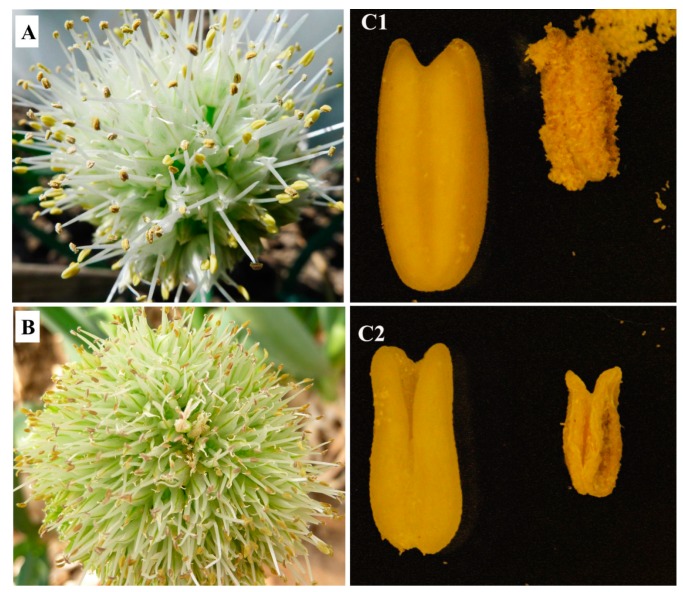
Comparison of cytoplasmic male maintainer and sterile varieties: (**A**) 64-1, cytoplasmic male maintainer; and (**B**) 64-2, cytoplasmic male sterility; (**C1**) 64-1 cytoplasmic male maintainer anther; and (**C2**) 64-2 cytoplasmic male sterility anther.

Two Welsh onion varieties (termed 64) were used in this study: 64-1, a CMS mutant in natural populations, and 64-2, a cytoplasmic male maintainer line, which when bred with 64-1 can produce fertile offspring (Figure 6). Both lines were bred at the Beijing Vegetable Research Center and the Beijing Academy of Agriculture and Forestry Sciences. For each variety, RNA was isolated from three inflorescences in the flowering stage of the Welsh onion in May 2013. We then collected experimental materials the same way in May 2014 as a repeat experiment. All samples were ground immediately after harvest in a mortar and pestle using liquid nitrogen. Total RNA was extracted using RNAiso for polysaccharide-rich Plant Tissue Kit (TaKaRa Biotechnology, Dalian, Liaoning Province, China), and RNA integrity was evaluated using an Agilent 2100 Bioanalyzer (Agilent Technologies, Santa Clara, CA, USA). RNA-seq was performed at Beijing BioMarker Technologies (Beijing, China). The paired-end library preparation, cDNA library and sequencing were performed following standard Illumina methods on the Illumina sequencing platform (HiSeq™ 2000). The read length of sequencing in both years was 2 × 103 bp.

All of the datasets from the Illumina sequencing platform can be found in the Short Read Archive (SRA) database of the National Center for Biotechnology Information (NCBI) under accession number SRP071555.

### 4.2. Sequence Data Analysis and Assembly

The Bcl2fastq software was used for pre-processing. The raw reads were cleaned by removing the adaptor sequences, duplication sequences, low-quality reads (reads with ambiguous bases “N”), and reads with more than 50% of the bases with a *Q*-value ≤5 [32] to obtain high-quality clean read data. The Trinity method [33] was used for de novo assembly. First, contigs were obtained by extension based on the overlap between sequences. Next, the resultant contigs were joined into transcripts with the paired-end information, and the following parameters were used: seqTypefq, min kmer cov 2, min contig length 200, group pairs distance 500 and other default parameters. The longest transcript was taken as the sample unigene, and the unigenes were combined to produce the final assembly used for annotation. Getorf [34] was used to predict the open reading frame (ORF) of the unigenes, and the longest ORF was extracted for each unigene. The Bowtie [35] was used to compare between unigene database and each sequencing read sample, and default parameters of the software were used.

### 4.3. Functional Annotation and Differential Gene Expression Analysis

The unigenes were aligned with the NCBI’s nucleotide (nt) databases and non-redundant (nr) protein, the Kyoto Encyclopedia of Genes and Genomes (KEGG) pathway database, the Swiss-Prot protein database, and the Cluster of Orthologous Groups (COG) database. The Blast2GO program was used to assign GO terms [36]. The BLAST [37] program was used to performed COG and KEGG pathway annotation. The above searches were performed with a cut-off *e*-value of 10^−5^. The unigenes were aligned to the COG and KEGG databases to predict and classify the possible functions and metabolic pathways of the unigenes. For differential gene expression analysis, Differentially expressed genes were identified using DESeq software [38,39] in each comparison, and the results were analyzed as described previously [1].

### 4.4. Quantitative RT-PCR (RT-qPCR) Analysis

The cDNA was synthesized from the flowering stage of the Welsh onion with total RNA in a 10-µL reaction system using PrimeScript II RTase (TaKaRa Biotechnology, Dalian, Liaoning Province, China). The reverse transcription reaction mixture contained 5 µL of total RNA (0.8 µg), 3 µL of diethylpyrocarbonate (DEPC) water, 1 µL of oligo dT (50 µM) and 1 µL of dNTP (10 mM), and the reaction was performed following the kit protocol. Three biological replicates and three technical replicates for each experiment were performed. The qPCR reactions were performed in 96-well plates on the LightCycler 480 instrument (Roche Diagnostic, Mannheim, Germany) using SYBR Green I (TaKaRa Biotechnology, Dalian, Liaoning Province, China). The qPCR primers, which were designed using Primer3 (http://frodo.wi.mit.edu/primer3/), are shown in Table S3. Each gene was analyzed using the method described previously by Qianchun Liu et al. [1].

## 5. Conclusions

This study investigated the important CMS-related genes and pathways in the Welsh onion by transcriptome analysis. Several key genes such as F-type ATPase, NADH dehydrogenase, and cytochrome *c* oxidase were observed by RNA-seq and validated by RT-qPCR. These genes were not only closely related to mitochondria but also to the nuclear–mitochondria interaction. Further functional characterization is in progress to determine the specific functions of these genes in relation to CMS in *Allium fistulosum* L. This study will accelerate the research on CMS gene clones and other functional genomics research on *A. fistulosum*.

## Figures and Tables

**Table 1:** Output statistics of sequencing analysis.

Sample	Total Reads	Total Nucleotides (bp)	GC Percentage	Q30 Percentage	Total Mapping Reads Percentage
CMS-2013	29,169,887	5,891,890,308	49.30%	87.75%	89.99%
CMM-2013	28,020,145	5,659,402,069	45.46%	89.18%	83.95%
CMS-2014	20,978,451	4,237,366,829	41.80%	95.07%	79.22%
CMM-2014	23,309,156	4,708,122,991	41.92%	95.11%	79.55%

CMS-2013: Cytoplasmic male sterility in May 2013; CMM-2013: Cytoplasmic male maintainer in May 2013; CMS-2014: Cytoplasmic male sterility in May 2014; CMM-2014: Cytoplasmic male maintainer in May 2014. Total mapping reads percentage: Total number of mapping reads percentage, including uniquely mapping reads and multiply mapping reads.

**Table 2:** Differentially expressed unigenes with significantly enriched pathways.

Pathway	DEGs with Pathway Annotation		All genes with Pathway Annotation		*p-*Value		Pathway ID
	May 2013	May 2014	May 2013	May 2014	May 2013	May 2014	
Plant-pathogen interaction	13	17	111	113	8.55 × 10^−5^	5.04 × 10^−5^	ko04626
	(9.35%)	(8.85%)	(2.73%)	(3.04%)			
Protein processing in endoplasmic reticulum	16	4	231	186	5.03 × 10^−3^	9.89 × 10^−1^	ko04141
	(11.51%)	(2.08%)	(5.69%)	(5%)			
Oxidative phosphorylation	13	15	171	161	5.24 × 10^−3^	1.76 × 10^−2^	ko00190
	(9.35%)	(7.81%)	(4.21%)	(4.33%)			
Starch and sucrose metabolism	13	15	173	152	5.77 × 10^−3^	1.08 × 10^−2^	ko00500
	(9.35%)	(7.81%)	(4.26%)	(4.08%)			
Plant hormone signal transduction	8	15	173	152	2.40 × 10^−1^	1.08 × 10^−2^	ko04075
	(5.76%)	(7.81%)	(4.26%)	(4.08%)			
Glycerophospholipid metabolism	5	10	84	90	1.59 × 10^−1^	1.64 × 10^−2^	ko00564
	(3.6%)	(5.21%)	(2.07%)	(2.42%)			

DEGs with pathway annotation: Figures in brackets represent the ratio of the number of DEGs that are annotated to this pathway and the number of DEGs that are annotated to all pathways. All genes with pathway annotation: Figures in brackets represent the ratio of all genes that are annotated to this pathway and all genes that are annotated to all pathways.

**Table 3:** The unigenes associated with Cytoplasmic male sterility (CMS).

Unigene ID	COG Class Annotation	KEGG Annotation	Swissprot Annotation	nr Annotation	nt Annotation
c116086	--	--	Plasma membrane ATPase (Wheat)	plasma membrane H^+^-ATPase (*Cucumissativus*)	PREDICTED: *Cucumissativus* plasma membrane ATPase 4-like (LOC101221564), mRNA >gi|449510556|ref|XM_004163650.1|
c175619	Energy production and conversion	K02261|1e-149|pop:POPTR_936218|hypothetical protein	Cytochrome c oxidase subunit 2 (Mouse-ear cress)	cytochrome oxidase subunit 2 (*Boehmerianivea*)	*Hibiscus cannabinus* cultivar P3B cytochrome c oxidase subunit II (*cox2*) gene, complete cds; mitochondrial
c159049	Energy production and conversion	K02262|0.0|ath:AT2G07687|cytochrome c oxidase subunit 3 (EC:1.9.3.1)	Cytochrome c oxidase subunit 3 (Wheat)	cytochrome oxidase subunit 3 (*Cliviaminiata*)	*Allium cepa* cultivar saski cytochrome oxidase subunit 2 (*cox2*) gene, exon 2 and partial cds; and cytochrome oxidase subunit 3 (*cox3*) gene, complete cds; mitochondrial
c160965	--	--	--	--	*Allium cepa* cultivar saski NADH dehydrogenase subunit 1 (*nad1*) gene, partial cds; and ATPase alpha subunit (*atpA*) gene, complete cds; mitochondrial
c113452	--	--	Probable F-box protein (Mouse-ear cress)	hypothetical protein MTR_139s0011 (*Medicagotruncatula*)	--
c50467	--	--	Polygalacturonase inhibitor 1 (Mouse-ear cress)	PREDICTED: polygalacturonase inhibitor-like (*Setariaitalica*)	--

--: No annotation information.

## Supplementary Materials

Supplementary materials can be found at http://www.mdpi.com/1422-0067/17/7/1058/s1.
