# Supplementary material for: Transcriptome Sequencing Analyses between the Cytoplasmic Male Sterile Line and Its Maintainer Line in Welsh Onion (Allium fistulosum L.)
Source: Int J Mol Sci. 2016 Jul 1;17(7):1058. doi: 10.3390/ijms17071058 (PMC4964434; doi:10.3390/ijms17071058)
Supplement: Supplementary file 1 [file ijms-17-01058-s001.pdf]

# Supplementary Materials: Transcriptome Sequencing Analyses between the Cytoplasmic Male Sterile Line and Its Maintainer Line in Welsh Onion (*Allium fistulosum* L.)

**Qianchun Liu, Yanping Lan, Changlong Wen, Hong Zhao, Jian Wang and Yongqin Wang**

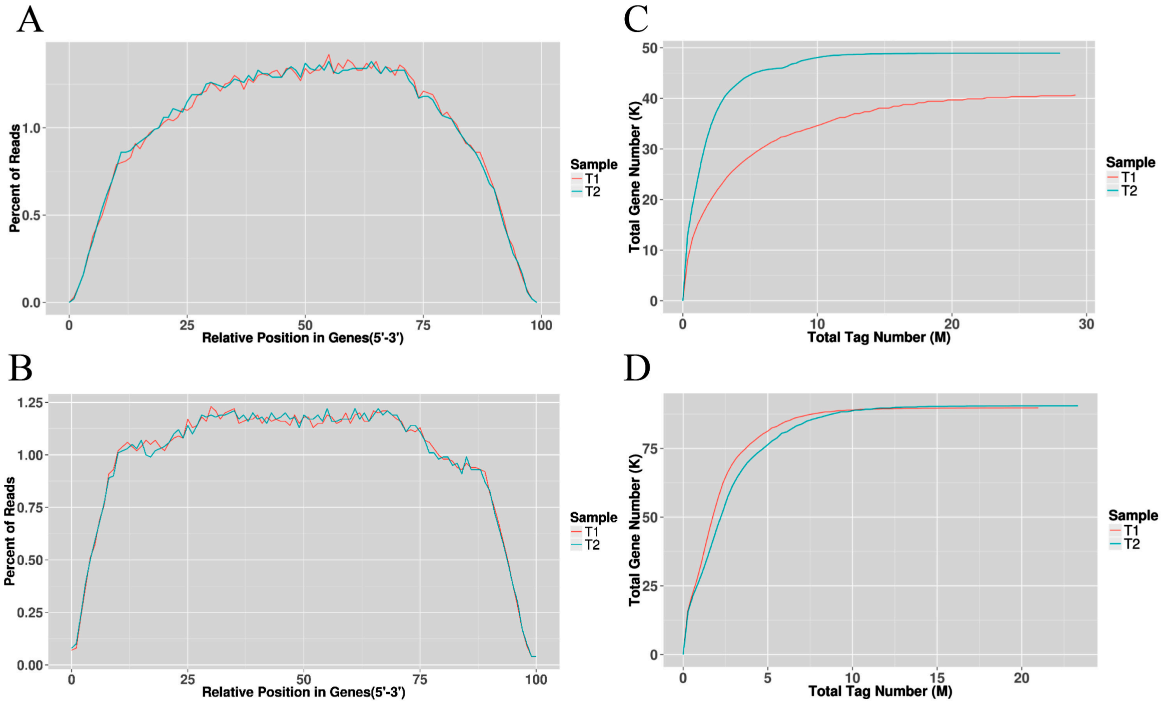


**Figure S1.** The sequencing quality testing of each sample: (**A**) Randomness test of cDNA fragments data from May 2013; (**B**) Randomness test of cDNA fragments data from May 2014; (**C**) Sequencing saturation analysis from May 2013; and (**D**) Sequencing saturation analysis from May 2014.

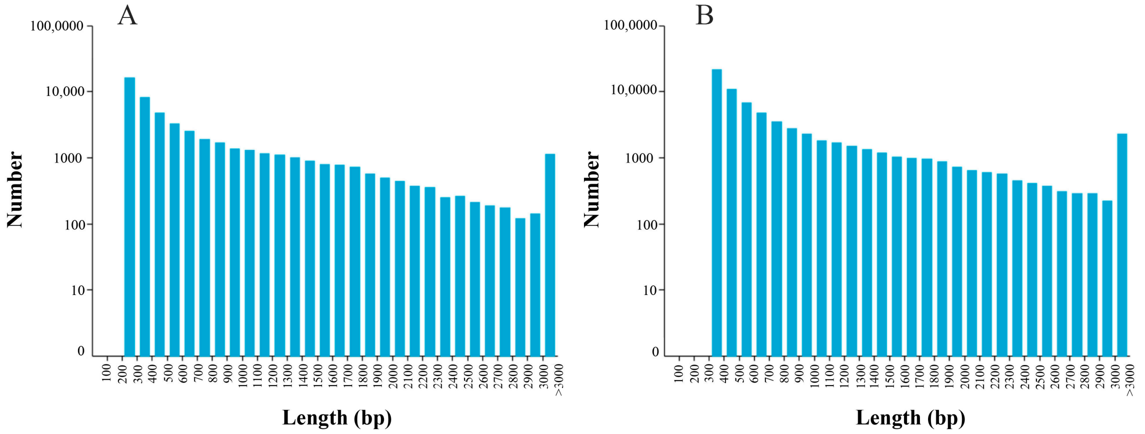


**Figure S2.** ORF (open reading frame) length distribution: (**A**) ORF length distribution data in May 2013; and (**B**) ORF length distribution data in May 2014.

**Table S1.** Length distribution of assembled contigs, transcripts, and unigenes.

| Nucleotide Length (bp) | Contigs | Transcripts | Unigenes |
|---|---|---|---|
| | May 2013/May 2014 | May 2013/May 2014 | May 2013/May 2014 |
| 200–300 | 2,473,748 (98.20%)/3,814,290 (97.60%) | 21,140 (21.74%)/57,966 (18.80%) | 16,261 (31.20%)/0 (0%) |
| 300–500 | 17,693 (0.70%)/43,811 (1.12%) | 20,653 (21.24%)/57,631(18.69%) | 12,799 (24.55%)/32,039 (45.54%) |
| 500–1000 | 14,321 (0.57%)/28,689 (0.73%) | 24,109 (24.80%)/68,847 (22.33%) | 10,660 (20.45%)/20,006 (28.43%) |
| 1000–2000 | 9866 (0.39%)/15,289 (0.39%) | 21,790 (22.41%)/73,238 (23.75%) | 8779 (16.84%)/11,948 (16.98%) |
| 2000+ | 3541 (0.14%)/6132 (0.16%) | 9538 (9.81%)/50,671 (16.43%) | 3627 (6.96%)/6367 (9.05%) |
| Total number | 2,519,169/3,908,211 | 97,230/308,353 | 52,126/70,360 |
| Total length | 150,905,138/287,324,074 | 89,214,332/346,171,877 | 39,390,976/61,896,802 |
| N50 length | 70/101 | 1419/1813 | 1247/1267 |
| Mean length | 59.90/73.51 | 917.56/1122.648 | 755.69/879.7158 |

**Table S2.** Functional annotation of unigenes.

| Annotation | Annotated Number | | DEGSs Number | |
|---|---|---|---|---|
| | May 2013 | May 2014 | May 2013 | May 2014 |
| Nr Annotation | 26,110 | 27,653 | 1057 | 1834 |
| Swissprot Annotation | 17,501 | 17,149 | 779 | 1296 |
| COG Annotation | 8326 | 8201 | 312 | 529 |
| GO Annotation | 20,599 | 17,541 | 819 | 1201 |
| KEGG Annotation | 5907 | 5423 | 184 | 284 |
| All Annotated | 26,262 | 29,491 | - | - |

**Table S3.** Primers used to perform qPCR of CMS-related gene biosynthesis and gene regulation.

| Unigene ID | Primer Sequence (5′to 3′) |
|---|---|
| c116086 | Forward: GGGTATCGTTGTGCTCCTGA<br>Reverse: CCCCATTTTCCATCTCTGAA |
| c175619 | Forward: TGCCATCATTTGTCTTTCCA<br>Reverse: GTGCTTTCAGGCTCTTCCAG |
| c159049 | Forward: ACTTCGCTCGCCTTCTGTC<br>Reverse: CGCCTCCTGCTTTCATAGAG |
| c160965 | Forward: CCGCTGCCAATACCAATAGT<br>Reverse: CTTCGCTTACACCGCTCAAT |
| c113452 | Forward: AGGTCTCTAAGGCAGGCAAA<br>Reverse: GGACTGAGGTGGCGAAATAC |
| c50467 | Forward: TCAAGCCGTCCAGTTTAGGA<br>Reverse: CCTCACCATCATCACCATCA |
| Actin | Forward: ACACGGCCTGGATAGCAACAT<br>Reverse: AGAGCAGTATTCCCAAGCATT |
